# Evaluation of Staining-Dependent Colour Changes in Resin Composites Using Principal Component Analysis

**DOI:** 10.1038/srep14638

**Published:** 2015-10-09

**Authors:** D. Manojlovic, L. Lenhardt, B. Milićević, M. Antonov, V. Miletic, M. D. Dramićanin

**Affiliations:** 1 University of Belgrade, School of Dental Medicine, Rankeova 4, Belgrade, 11000, Serbia; 2 University of Belgrade, Vinča Institute of Nuclear Sciences, P.O. Box 522, Belgrade, 11001, Serbia

## Abstract

Colour changes in Gradia Direct™ composite after immersion in tea, coffee, red wine, Coca-Cola, Colgate mouthwash, and distilled water were evaluated using principal component analysis (PCA) and the CIELAB colour coordinates. The reflection spectra of the composites were used as input data for the PCA. The output data (scores and loadings) provided information about the magnitude and origin of the surface reflection changes after exposure to the staining solutions. The reflection spectra of the stained samples generally exhibited lower reflection in the blue spectral range, which was manifested in the lower content of the blue shade for the samples. Both analyses demonstrated the high staining abilities of tea, coffee, and red wine, which produced total colour changes of 4.31, 6.61, and 6.22, respectively, according to the CIELAB analysis. PCA revealed subtle changes in the reflection spectra of composites immersed in Coca-Cola, demonstrating Coca-Cola’s ability to stain the composite to a small degree.

Colour changes in restorative composites upon exposure to simulated oral environments have been the subject of extensive research in recent years. Many materials have been immersed in staining agents, most frequently drinks and mouthwashes, and their colour changes have been quantified and analysed^1^,^2^,^3^,^4^,^5^,^6^,^7^,^8^,^9^,^10^,^11^,^12^,^13^. Because the colours of materials can be expressed with coordinates in colour spaces (usually in the Commission International de l’Eclairage colour system – CIE or Munsell Colour System), variations in the values of colour coordinates can be considered as quantifiers of the colour changes. For example, the quantifiers in the CIELAB colour space are the differences in the lightness (ΔL*), intensities, and directions of the green-red coordinate (Δa*) and the blue-yellow coordinate (Δb*) as well as the total change in colour (ΔE = [(ΔL*)^2^ + (Δa*)^2^ + (Δb*)^2^]^1/2^) and chroma (ΔC = [(Δa*)^2^ + (Δb*)^2^]^1/2^). The effects of various parameters in a staining process, such as the type and concentration of the agent, the duration of exposure, and the quality of the material surface have been evaluated by descriptive and/or statistical analyses of the colour change quantifiers^14^,^15^,^16^,^17^,^18^,^19^,^20^,^21^.

However, it should be noted that the description of optical properties using colour space coordinates is performed after compressing the data, which results in a substantial amount of valuable information about the material’s surface being hidden or lost. Consequently, a colour-coordinate–based analysis may lack some information to accurately address and explain a range of staining effects on restorative materials. Considering that staining processes affect the surface reflectance of the material, we believe that an analysis on the staining of dental restorative materials and teeth should focus on changes in surface reflectance after staining.

Two issues are key to analysing the surface reflection of materials to characterize staining. First, it is important to conclusively establish whether the reflection is changed after the exposure of the material to staining agents. If the magnitude of the change in reflection is negligible or within the boundaries of the measurement error, no colour changes in the material can be argued. Second, the analysis should expose the parts of the reflection spectrum affected by staining. Then, it is possible to discover colorant species in the staining solution that contribute to discoloration and to assess the scale of their staining ability. Both issues can be simultaneously addressed using principal component analysis (PCA), which is a well-known multivariate statistical method. This method transforms and compresses many possibly correlated variables into a smaller number of uncorrelated variables called principal components (PCs), which account for most of the variance in the observed variables^22^. In colour science, this method has been successfully used for many applications^23^. For analysis of reflection, PCA can be used to consider the complete reflection spectra of individual objects for calculations. In this approach, the input data consist of a series of reflection coefficient values assigned to objects that are divided into groups. The groups consist of unstained materials and materials exposed to staining agents.

In this report, we present the results obtained using the approach described above to analyse staining of the microhybrid composite Gradia Direct, extra bleach white (XBW) shade. PCA was applied to diffuse reflectance spectra of material samples exposed to the following common staining agents: tea, coffee, red wine, Coca-Cola, Colgate mouthwash, and distilled water. The spectra were compared to samples before staining. The observations from the PCA were corroborated by colour change results calculated using the CIELAB colour system. The null hypotheses were: (*i*) PCs and scores from the PCA model do not present conclusive information about whether statistically significant differences exist between the reflection spectra of materials before and after staining; (*ii*) PC loadings cannot reveal the parts of the reflection spectra that contribute most to differences between groups.

## Results and Discussion

**Figure 1:**
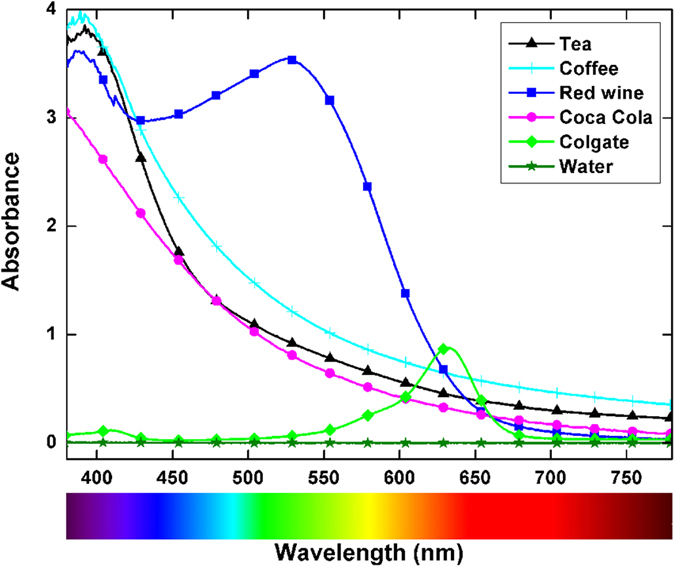
Absorption spectra of the staining solutions in the 380–780 nm range.

Composite samples were immersed in staining solutions having the absorption spectra shown in Fig. 1. The tea, coffee, red wine, and Coca-Cola solutions showed strong absorption in the 380–500 nm spectral range. Red wine showed an additional strong absorption band centred at approximately 530 nm. The Colgate solution had lower absorption compared to the other staining solutions, with its main absorption band centred at 630 nm. Distilled water, as a control, showed no absorption.

**Figure 2:**
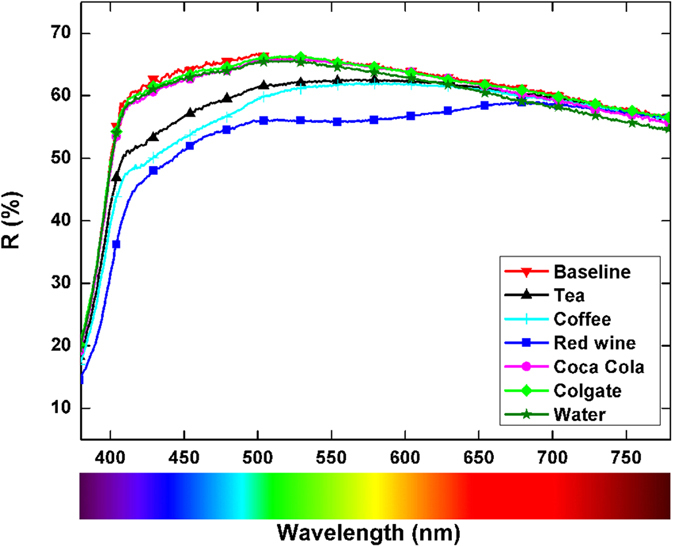
Reflection spectra of the baseline composite and the samples stained in the different solutions.

The diffuse reflectance spectra of composite samples before (baseline) and after staining are presented in Fig. 2. The spectra were obtained after averaging the spectra of multiple samples from the same group. The spectra of samples stained in coffee, red wine, and tea appeared different than the spectra of the samples stained in the other solutions. Subtle differences in the reflectance spectra of the samples stained in Coca-Cola and the Colgate solution were observed in the 400–550 nm spectral range compared to the baseline group.

### Results of PCA

**Figure 3:**
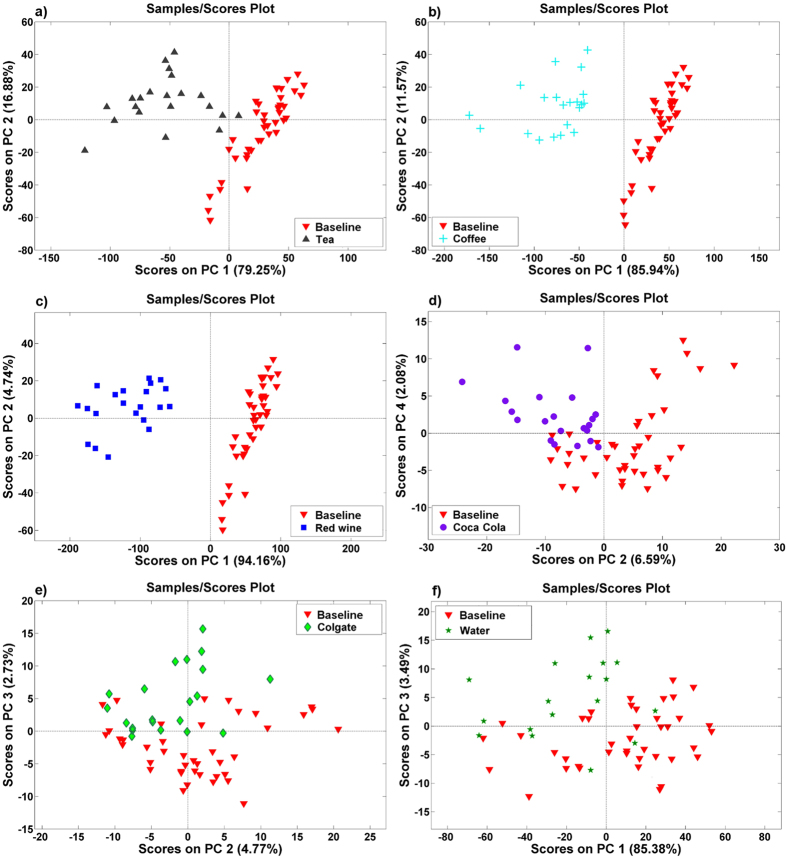
PCA score plots of the composite samples stained in (**a**) tea, (**b**) coffee, (**c**) red wine, (**d**) Coca-Cola, (**e**) Colgate mouthwash, and (**f**) distilled water relative to the baseline group.

**Figure 4:**
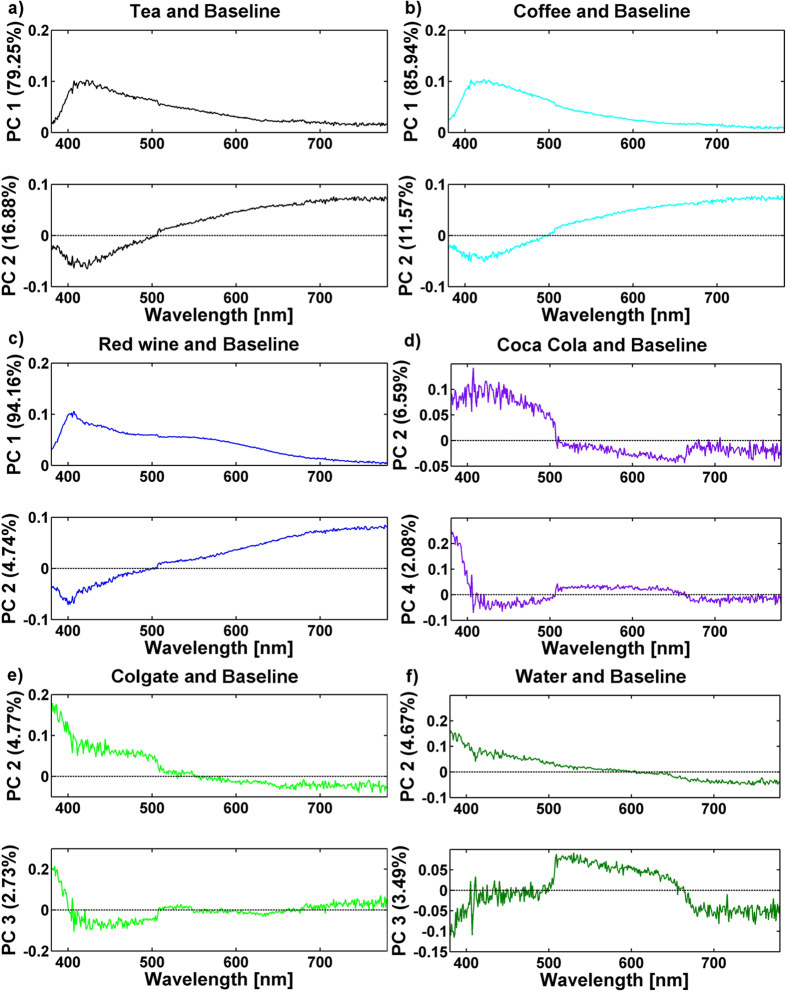
Loadings of the original data (diffuse reflection coefficients) to PCs from PCA composite samples stained in (**a**) tea, (**b**) coffee, (**c**) red wine, (**d**) Coca-Cola, (**e**) Colgate mouthwash, and (**f**) distilled water relative to the baseline group.

Figure 3(a–f) presents PCA score plots for the six sample groups: tea, coffee, red wine, Coca-Cola, Colgate mouthwash, and distilled water, respectively, compared to the baseline group. Similarly, Fig. 4(a–f) show the original data (diffuse reflection coefficients) loaded into the PCs.

In Fig. 3(a–c), a clear separation can be observed between the groups of samples stained with tea, coffee, and red wine. The first two PCs accumulated 96.13%, 97.51%, and 98.90% of the variance of the diffuse reflection spectra for tea, coffee, and wine, respectively. It is thus possible to conclude that the diffuse reflection spectra of the composite are significantly changed after staining in these solutions. The loadings display diffuse reflection data that varied the most between the stained and as-prepared samples (Fig. 4(a–c)). For tea, the largest difference was observed for the data measured in the 380–450 nm range, and this difference correlated well with the absorption spectrum of tea (Fig. 1), where the absorption comes mainly from tannins^24^. For coffee, the spectral range with the most pronounced differences compared to the baseline group was similar to the case of tea. Tea and coffee have similarly shaped absorption spectra (Fig. 1); the major difference is in the magnitude of absorption, which is higher in coffee than in tea. The absorption of coffee in the 380–780 nm spectral range is mainly governed by the absorption of melanoidins^25^. These high-molecular-weight nitrogenous and brown-coloured compounds are formed during coffee roasting as the final products of the Maillard reaction. The largest difference between the diffuse reflection spectra of the stained and baseline samples was observed for the red wine solution. The magnitude of the difference between the reflection spectra is shown in Fig. 2, and 98.90% of the variance was accumulated in the first two PCs (Fig. 3(c)). The reflection spectra of the composite samples stained in the red wine solution changed over a wide spectral range, from 380 to 700 nm; however, the largest change occurred in the green portion of the visible spectra (Fig. 4(c)), giving the human eye the sensation of a red colour. The anthocyan complexes in red wine are responsible for this effect^26^.

As shown in Fig. 2, changes in the reflection spectra were barely visible for composites stained with Coca-Cola, Colgate mouthwash, and distilled water. In addition, the PCA score plots, which are shown in Fig. 3(d–f), did not show a clear distinction between the stained groups and baseline samples. Therefore, it is reasonable to conclude that no significant changes in the reflection spectra occur after 3-days of staining with Coca-Cola, Colgate mouthwash, or distilled water; hence, no changes in the composite’s colour can be claimed. Among the PCA results of these three staining solutions, the PCA scores for Coca-Cola revealed potential separation between groups (Fig. 3(d)). The difference was observed with the second and fourth PC, which accumulated 8.67% of the total variance between the groups. The PCA input for this case, shown in Fig. 4(d), was able to reveal subtle changes (approximately 10% change in the second PC and 20% change in the fourth PC) in the reflection spectra of the composites exposed to Coca-Cola in the 380–500 nm range; it should be noted that Coca-Cola exhibits absorption in this spectral region (Fig. 2) because of the presence of sulphite ammonia caramel (E150d dye)^27^. This result indicates that some discoloration processes are occurring but that they are on a considerably smaller scale than those occurring with tea, coffee, and red wine. Absorption of the synthetic dye Brilliant Blue FCF (E133) is responsible for the colour of the Colgate solution^28^. However, the absorbance value of Colgate mouthwash at 627 nm indicates a relatively low concentration of the dye, at least compared to the absorbance of colorant species in the other staining solutions; therefore, no significant staining of the composites was expected during 3-day immersion in this solution. Considering the ability of PCA to present conclusive information on the discoloration of composites after exposure to staining agents, as well as its ability to reveal differences in the reflection spectra, the null hypotheses were rejected,

### Discoloration effects from colour quantifiers in the CIELAB colour space and their relationship to the PCA results

The colour coordinates of the composite samples were calculated from their corresponding diffuse reflection spectra for the standard illuminant D65 (*Standard daylight*). The mean values of the colour coordinates for the different sample groups (baseline and stained in tea, coffee, red wine, Coca-Cola, Colgate mouthwash, and distilled water) are listed in Table 1.

Before staining, the composite samples were white and bright (L*~ 84.5) with a small amount of green and yellow shades. The main changes in the diffuse reflection spectra (Fig. 2.) originated from colorant species that absorb in the blue spectral region. Therefore, one can observe a general trend of the b* value increasing for all the staining solutions due to reduction of the blue colour. Therefore, the composites appeared yellowish-reddish after staining. The total differences in the colour and chroma as well as differences in the composite’s lightness, green-red coordinate, and blue-yellow coordinate after exposure to the staining solutions are presented in Table 2.

The total colour change was significant in the composites exposed to staining in tea, coffee, and red wine, whereas the changes upon exposure to Colgate mouthwash and distilled water were negligible. The total colour change in the samples exposed to Coca-Cola was near the detection limit (∆E < 1 is barely recognizable^29^,^30^,^31^,^32^; ∆E ≤ 2.7 is a clinically acceptable difference in colour^33^). For staining in tea and coffee, the main contribution to the total colour change came from the change of chroma. For red wine, changes in chroma and lightness contributed to the total colour change to approximately the same extent. The colours of samples stained in tea, coffee, and red wine were measured again after desorption in distilled water for 7 days. The total colour change of the samples decreased 12.7% in the case of composites stained in coffee, 15.7% for those stained in wine, and 28% for those stained in tea. The colour change observations for the CIELAB colour coordinates correlated well with those from the PCA. Considering that both the PCA and analysis from CIELAB coordinates revealed slight changes in the diffuse reflection spectrum and colour coordinates of the composites exposed to Coca-Cola, it can be concluded that the Coca-Cola solution exhibits staining ability. One should note that the use of a single shade of a single product in the presented research precludes generalization of the findings on the extent of staining-dependent changes in the colour of resin composites, especially because the shade used was extra light, likely making it more susceptible to discoloration. However, the aim of this study was to evaluate the utility of PCA in colour analysis for the staining of resin composites, and the shade used was utilized as a model material.

## Conclusion

Analysis of the reflection spectra provided information on the level and nature of the staining-dependent changes in the colour of resin composites. PCA of the composites’ reflection spectra produced conclusive information on the appearance of discoloration in samples immersed in different staining solutions. In the presented case, PCA scores conclusively showed that coffee, tea and red wine induce discoloration of the composites and that Colgate mouthwash and distilled water do not; low-level discoloration was found for samples immersed in Coca-Cola. Furthermore, PCA loadings showed which parts of the reflection spectra were affected by staining and to which extent, thereby revealing the species responsible for staining of the resin composites to be tannins (tea), melanoidins (coffee), anthocyanes (red wine), and sulphite ammonia caramel (Coca Cola).

As a general trend, the reflection spectra of the stained samples exhibited lower reflection in the blue spectral region, which was manifested in the reduced blue shade in the sample colour. This effect was much more pronounced in the samples exposed to tea, coffee, and red wine compared to the other solutions. Consequently, the values of CIELAB b* colour coordinate increased for all the stained samples. For staining in tea and coffee, the main contributor to the total colour change was the change of chroma. For red wine, changes in chroma and lightness contributed to the total colour change to approximately the same extent. Colgate mouthwash and distilled water produced discoloration that was lower than the perception limit, and Coca-Cola resulted in a total colour change that was just above the perception limit.

## Methods

### Sample preparation

A total of 168 samples of microhybrid composite Gradia Direct™ XBW shade (GC Corp. Tokyo, Japan), 13 mm in diameter and 1.5-mm thick, were prepared in silicon moulds held between two glass slides. The samples were cured with a polywave LED light-curing unit (bluephase G2, IvoclarVivadent, Schaan, Liechtenstein; 1100 mW/cm^2^ light intensity) for 20 s. Light-curing was performed through a 1 mm glass slide to maintain a constant distance. The light intensity was monitored with a bluephase metre (IvoclarVivadent, Schaan, Liechtenstein). Immediately after polymerization, the samples were removed from the mould and polished with 600- and 1200-grit abrasive discs (Buehler, Lake Bluff, IL, USA) under wet conditions. Subsequently, all the samples were stored in distilled water at 37 °C for 24 h. A total of 42 samples were used as a reference group (baseline). The remaining samples (126) were exposed to staining solutions.

### Staining solutions and sample staining

Six different staining solutions were prepared: tea (Black tea, English breakfast, Sir Winston company LTD, London, UK; a prefabricated tea bag was immersed in 150 ml of boiling water for 5 min according to the manufacturer’s instructions), coffee (Nescafe Classic, Nestle, Vevey, Switzerland; 3 g coffee powder was dissolved in 150 ml of boiling water), cola (Coca-Cola HBC, Zemun, Serbia), Colgate mouthwash (Colgate® plax cool mint, Colgate-Palmolive AG, Therwil, Switzerland), red wine (J. P. Chenet Cabernet Syrah, Petersbach, France), and distilled water. Samples were randomly selected and divided into six groups of 21 samples each to be exposed to the six staining solutions. Each sample was immersed in 15 ml of staining solution and stored at 37 °C. The staining solutions were renewed daily to prevent bacterial contamination. After 3 days, the samples were rinsed with tap water for 10 s and blot-dried with paper towels.

### Diffuse reflection measurements

Diffuse reflection measurements of the samples were performed in the 380–780 nm range with a 1-nm step size using a Thermo Evolution 600 spectrophotometer (Thermo Fisher Scientific, Waltham, MA, USA) equipped with an integrating sphere (Labsphere RSA-PE-19). Barium sulphate was used as the reference material.

### PCA

PCA was performed six times for the groups of samples exposed to the different staining solutions versus the group of baseline samples. PCA was performed from the dataset matrices (i.e., 63 samples × 401 coefficients of reflection) using the singular value decomposition method and a standard procedure^34^, which did not require any computational parameter settings. Prior to analysis, the data were mean centred. Each PCA resulted in a score and loading matrices. The score matrix (i.e., 63 samples × the number of PCs) contained the coordinates of every sample in the new PC coordinate system. The loading matrix (i.e., the number of PCs × 401 coefficients of reflection) indicated the contribution of every original variable to the PCs. The percentage of cumulative variance captured in the original data for every PC was calculated. All the calculations were performed using Solo Version 6.5.4 software (Eigenvector, 108 Inc., Chelan, WA, USA).

The colour coordinates were calculated in the CIELAB colour system from diffuse reflection spectra for D65 illuminant (Standard Daylight). The extent of colour changes observed under different illuminants (D65 illuminant (Standard Daylight), A (Incandescent/Tungsten), D50 (Mid-morning/afternoon Daylight), D75 (North Sky Daylight), CW-Fluo (Cool White Fluorescent), WW-Fluo (Warm White Fluorescent), and HP (High-Pressure Lamp)) are presented in the Supplement file.

## Additional Information

**How to cite this article**: Manojlovic, D. *et al*. Evaluation of Staining-Dependent Colour Changes in Resin Composites Using Principal Component Analysis. *Sci. Rep*. **5**, 14638; doi: 10.1038/srep14638 (2015).

## Supplementary Material

Supplementary Information

## Figures and Tables

**Table 1:** Mean values of CIELAB colour coordinates for baseline composite samples and samples stained in various solutions.

	L*	a*	b*
Baseline	84.49	−2.22	0.91
Tea	82.93	−2.22	4.92
Coffee	82.46	−2.64	7.19
Red wine	79.66	−1.36	4.73
Coca-Cola	84.49	−2.71	2.10
Colgate	84.47	−2.68	1.58
Water	84.06	−2.76	1.35

**Table 2:** Total change in colour (∆E), chroma (∆C), lightness (∆L*), green-red coordinate (Δa*), and blue-yellow coordinate (Δb*) for composite samples stained in various solutions and compared to baseline samples.

	∆E	∆C	∆L*	∆a*	∆b*
Tea	4.31	4.02	1.56	0.00	−4.02
Coffee	6.61	6.29	2.03	0.42	−6.28
Red wine	6.22	3.92	4.83	−0.86	−3.82
Coca-Cola	1.29	1.29	0.00	0.48	−1.19
Colgate	0.89	0.82	0.02	0.46	−0.68
Water	0.82	0.70	0.43	0.54	−0.44
