# Supplementary material for: Evaluation of Staining-Dependent Colour Changes in Resin Composites Using Principal Component Analysis
Source: Sci Rep. 2015 Oct 9;5:14638. doi: 10.1038/srep14638 (PMC4598812; doi:10.1038/srep14638)
Supplement: Supplementary Information [file srep14638-s1.pdf]

Evaluation of Staining-Dependent Colour Changes in Resin Composites Using Principal Component Analysis

D. Manojlovic[1], L. Lenhardt[2], B. Milićević[2], M. Antonov[2], V. Miletic[1] & M. D. Dramićanin[2*]

[1] University of Belgrade, School of Dental Medicine, Rankeova 4, Belgrade, 11000, Serbia

[2] University of Belgrade, Vinča Institute of Nuclear Sciences, P.O. Box 522, Belgrade, 11001, Serbia

*Corresponding author email: dramican@vinca.rs

Supplement 1: Extent of colour changes observed under different illuminants.

The spectral characteristics of illuminants play an important role in the perception of colour (Figure S1). The coloration of composite samples appears slightly different under different illuminants. The total colour changes and changes in chroma of samples under illuminants A, D50, D75, CW-Fluo, WW-Fluo, and HP compared with the standard illuminant D65 are shown in Table S1. The colour of the samples did not vary much among the daylight illuminants D65, D50, and D75 (the total change of colour is lower than 1). The largest colour difference was observed between the incandescent (A) and high-pressure lamp (HP) illuminants. For the red wine and coffee stained composites the total colour change was ~ 2, and for the samples stained in tea the change was ~1.5. For the rest of the groups the change was ~1, which is near the perception limit. The colour difference observed with a warm white fluorescent lamp (WW-Fluo) compared to the D65 illuminant was higher than 1 for all the groups (the largest difference was observed for coffee staining, ~ 2.3), whereas for a cool white fluorescent lamp (CW Fluo) the changes were smaller than 1, with the exception of the coffee-stained group, which had a total colour change of ~1.2. Nearly all of the total colour change was attributed to the change in chroma, as can be observed by comparing the data in Table S1.

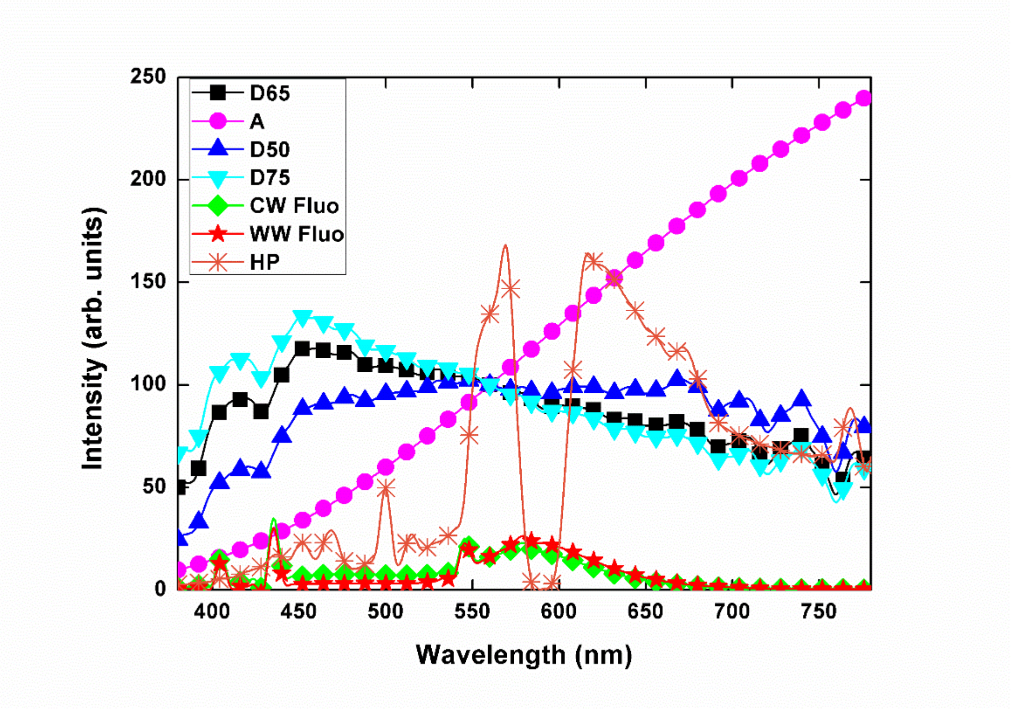

Figure S1. Spectral characteristics of standard illuminants: D65 (Standard Daylight), A (Incandescent/Tungsten), D50 (Mid-morning/afternoon Daylight), D75 (North Sky Daylight), CW-Fluo (Cool White Fluorescent), WW-Fluo (Warm White Fluorescent), and HP (High-Pressure Lamp).

Table S1. Total changes in colour and chroma of composite samples after staining measured under different illuminants and compared with the standard illuminant D65.

	A		D50		D75		CW Fluo		WW Fluo		HP	
	ΔE	ΔC	ΔE	ΔC	ΔE	ΔC	ΔE	ΔC	ΔE	ΔC	ΔE	ΔC
**Baseline**	0.76	0.74	0.24	0.24	0.12	0.11	0.71	0.71	1.02	1.00	0.80	0.77
**Red wine**	2.05	2.04	0.82	0.82	0.42	0.42	0.81	0.81	1.79	1.79	2.25	2.24
**Water**	0.86	0.84	0.25	0.25	0.12	0.12	0.90	0.89	1.27	1.25	0.96	0.92
**Tea**	1.67	1.66	0.67	0.67	0.36	0.36	0.98	0.97	1.77	1.76	1.58	1.57
**Colgate**	0.90	0.88	0.28	0.28	0.14	0.14	0.85	0.85	1.24	1.23	0.99	0.95
**Coffee**	2.23	2.22	0.91	0.91	0.48	0.48	1.22	1.20	2.23	2.21	2.09	2.07
**Coca-Cola**	0.97	0.96	0.32	0.32	0.16	0.16	0.88	0.88	1.31	1.00	1.00	0.98
